# Differential expression of E-type prostanoid receptors 2 and 4 in microglia stimulated with lipopolysaccharide

**DOI:** 10.1186/s12974-016-0780-7

**Published:** 2017-01-05

**Authors:** Ester Bonfill-Teixidor, Amaia Otxoa-de-Amezaga, Miriam Font-Nieves, M. Glòria Sans-Fons, Anna M. Planas

**Affiliations:** 1 Departament d’Isquèmia Cerebral i Neurodegeneració, Institut d’Investigacions Biomèdiques de Barcelona (IIBB), Consejo Superior de Investigaciones Científicas (CSIC), Barcelona, Spain; 2 Institut d’Investigacions Biomèdiques August Pi i Sunyer (IDIBAPS), Barcelona, Spain; 3 Department of Brain Ischemia and Neurodegeneration, Institut d’Investigacions Biomèdiques de Barcelona (IIBB), Consejo Superior de Investigaciones Científicas (CSIC), Rosselló 161 planta 6, 08036 Barcelona, Spain

**Keywords:** PGE_2_, EP4, EP2, COX-2, Neuroinflammation, Glia, Mice

## Abstract

**Background:**

Cyclooxygenase-2 (COX-2) is induced under inflammatory conditions, and prostaglandin E_2_ (PGE_2_) is one of the products of COX activity. PGE_2_ has pleiotropic actions depending on the activation of specific E-type prostanoid EP1-4 receptors. We investigated the involvement of PGE_2_ and EP receptors in glial activation in response to an inflammatory challenge induced by LPS.

**Methods:**

Cultures of mouse microglia or astroglia cells were treated with LPS in the presence or absence of COX-2 inhibitors, and the production of PGE_2_ was measured by ELISA. Cells were treated with PGE_2_, and the effect on LPS-induced expression of TNF-α messenger RNA (mRNA) and protein was studied in the presence or absence of drug antagonists of the four EP receptors. EP receptor expression and the effects of EP2 and EP4 agonists and antagonists were studied at different time points after LPS.

**Results:**

PGE_2_ production after LPS was COX-2-dependent. PGE_2_ reduced the glial production of TNF-α after LPS. Microglia expressed higher levels of EP4 and EP2 mRNA than astroglia. Activation of EP4 or EP2 receptors with selective drug agonists attenuated LPS-induced TNF-α in microglia. However, only antagonizing EP4 prevented the PGE_2_ effect demonstrating that EP4 was the main target of PGE_2_ in naïve microglia. Moreover, the relative expression of EP receptors changed during the course of classical microglial activation since EP4 expression was strongly depressed while EP2 increased 24 h after LPS and was detected in nuclear/peri-nuclear locations. EP2 regulated the expression of iNOS, NADPH oxidase-2, and vascular endothelial growth factor. NADPH oxidase-2 and iNOS activities require the oxidation of NADPH, and the pentose phosphate pathway is a main source of NADPH. LPS increased the mRNA expression of the rate-limiting enzyme of the pentose pathway glucose-6-phosphate dehydrogenase, and EP2 activity was involved in this effect.

**Conclusions:**

These results show that while selective activation of EP4 or EP2 exerts anti-inflammatory actions, EP4 is the main target of PGE_2_ in naïve microglia. The level of EP receptor expression changes from naïve to primed microglia where the COX-2/PGE_2_/EP2 axis modulates important adaptive metabolic changes.

**Electronic supplementary material:**

The online version of this article (doi:10.1186/s12974-016-0780-7) contains supplementary material, which is available to authorized users.

## Background

Cyclooxygenase-2 (COX-2) is induced in brain cells under inflammatory conditions, but the role of COX-2 in the inflammatory response is very complex because it participates in different stages from initiation to resolution. While COX-2 is associated to inflammation and COX-2 inhibitors have anti-inflammatory properties, COX-2-deficient mice show exacerbated inflammation, leukocyte infiltration, and blood-brain barrier damage after exposure to the bacterial lipopolysaccharide (LPS), while these effects are attenuated in COX-1-deficient mice [1–5]. The COX pathway generates multifunctional vasoactive prostanoids in a cell type-dependent manner depending on the relative expression of constitutive COX-1 and inducible COX-2 and on the preferential coupling of COX isoforms with prostanoid synthases [6]. Prostaglandin E_2_ (PGE_2_) is a main product of COX-2 activity in glial cells under inflammatory conditions [7]. PGE_2_ exerts pro- (e.g., [8–10]) and anti-inflammatory (e.g., [11–13]) actions likely due to its involvement in regulating the onset, course, magnitude, and duration of the inflammatory response [14]. Furthermore, PGE_2_ plays crucial actions in regulating immune responses [15].

PGE_2_ exerts pleiotropic effects depending on signaling through four distinct E-type prostanoid (EP) receptors: EP1–4 [16]. EP receptors are G-protein-coupled plasma membrane receptors activating different signal transduction pathways [17]. Therefore, the type of response to PGE_2_ depends on the cell/tissue-specific expression of the EP receptors. EP4 selective agonists decrease LPS-induced pro-inflammatory gene expression in mouse microglia [18]. In cultured rat microglial cells exposed to LPS and IFN-γ, PGE_2_ induces complex effects, including the up-regulation of COX-2, iNOS, IL-6, and IL-1β but down-regulation of TNF-α, and all these effects were attributed to EP2 [19]. Here, we investigated how COX-2, PGE_2_, and EP receptors affected the dynamic response of cultured glial cells to the pro-inflammatory stimulus of LPS.

## Methods

### Cell cultures

Primary cell cultures of glial cells were obtained from postnatal C57BL/6J mice. Glial cell cultures enriched in astrocytes were prepared from the cerebral cortex of 1- to 2-day-old mice, as previously described [20, 21], with minor modifications. In brief, the cells were maintained at 37 °C in a humidified atmosphere of 5% CO_2_-95% air in complete culture medium: DMEM:F-12 nutrient (1:1) (Gibco-BRL), supplemented with 10% fetal bovine serum (FBS; Gibco-BRL) and 4 mL/L of a mixture of penicillin/streptomycin 10,000 U/10,000 μg/mL (Gibco-BRL). Cells were subcultured to obtain purified astroglia cultures, as follows: at confluence after 8–10 days in vitro, cells were treated with 4 μM of the antimitotic cytosine arabinoside (Ara-C, Sigma-Aldrich) for 2 days to eliminate dividing cells, i.e., mostly microglia and progenitors; then, flasks were shaken overnight, and the remaining astrocyte adherent monolayer was detached with trypsin 0.0125%/EDTA 1 mM and seeded at 10 × 10^4^ cells/mL with an incubation medium (as above) on polylysine-coated plates. Purified astrocytes were treated when cells reached confluence at 4 days after subculturing. FBS was reduced to 1% for 16 h prior to treatments. Astrocyte cultures contained only 2% of contaminating microglia cells [7, 21]. Highly pure microglia cell cultures were prepared following the *mild trypsinization* method described previously [22], with minor modifications. Briefly, mixed glia cultures were maintained 19 days in vitro, performing a subculture to increase the efficiency at day 8, as described above. Astrocyte monolayer was discarded and bottom microglia was kept, as follows: the cells were incubated for 30 min with trypsin 0.0625%/EDTA 1 mM causing the detachment of an upper layer of astrocytes in one piece. The remained attached microglia was maintained in a culture medium solution containing half medium of mixed glia cultures and half new culture medium. Purified microglia was treated 1 day after purification with reduction of FBS to 1% 1 h prior to treatments. Microglia culture purity was determined by counting the number of isolectin-positive cells out of the total cell nuclei number per area in four different areas (×20 objective) in four independent microglia cultures. The mean ± SD percentage of microglial cells was 97 ± 2.8% (see Additional file 1: Figure S1).

Primary cultures of macrophages were obtained from the bone marrow of adult (3 months old) male C57BL/6 mice. The cells were cultured in DMEM containing 10% FBS, penicillin/streptomycin as above, and 30% L-Cell medium obtained from the L929 cell line. After 6 days in culture, macrophages were replated (250,000 cells/mL). The following day, the medium was replaced by DMEM with 1% FBS, and cells were treated 1 hour later.

### Drug treatments

The cells were exposed to LPS (*Escherichia coli* 055:B5) (Sigma-Aldrich, St. Louis, MO, USA) (10 ng/mL, unless otherwise stated). The following COX-2 inhibitors were used: 3 μM N-[cyclohexyloxy-4-nitrophenyl] methanesulfonamide (NS-398; Tocris Bioscience, Ellisville, MO, USA), 10 μM celecoxib and 2,5-dimethyl-celecoxib inactive analog (Sigma-Aldrich), 10 nM sc-791-COX2 Inhibitor II (Calbiochem, EMD Millipore, Merck KGaA, Darmstadt, Germany), and 10 nM CAY 10404 (Cayman Chemical Co., Ann Arbor, MI, USA). Drug inhibitors were dissolved in dimethyl sulfoxide (DMSO). Prostaglandin E_2_ (PGE_2_) (1.4–11.3 nM in ethanol) was from Sigma-Aldrich. The EP4 agonist ONO-4819 (100 nM in ethanol) and EP2 agonist butaprost (1 μM in DMSO) were from Cayman Chemical Co. Selective EP receptor antagonists (Tocris Bioscience) were used: EP1 antagonist (SC 51089, 5 μM), EP2 antagonist (PF 04418948, 1 μM), EP3 antagonist (L-798,106, 0.5 μM) and EP4 antagonist (GW 627368, 1 μM). EP antagonists were dissolved in DMSO. Drugs were diluted in phosphate-buffered saline (PBS). The final ethanol or DMSO concentration did not exceed 0.0005 or 0.00015%, respectively. Corresponding vehicles were used in all experiments to check for non-specific effects. The above drug concentrations correspond to the final concentration in the culture medium. Drug concentrations were chosen based on the half maximal inhibitory concentration, literature reports, and preliminary experiments carried out in primary cultures of macrophages and microglia (see Additional file 2: Figure S2).

### Western blotting

Cells were lysed in radioimmunoprecipitation assay (RIPA) buffer containing protease inhibitors. Five micrograms of protein were resolved by SDS-PAGE, and the proteins were transferred to polyvinylidene difluoride membranes. Rabbit polyclonal antibodies were used against vascular endothelial growth factor-A (VEGFA) (#ab46154, Abcam) diluted 1:500; NADPH oxidase 2 (NOX2/gp91phox) (#ab129068, Abcam) diluted 1:500; and EP2 receptor (#APR-064, kindly provided by Alomone Labs, Jerusalem, Israel) diluted 1:1000. Mouse monoclonal antibodies against β-tubulin (#T4026, Sigma, St. Louis, MO, USA) (1:10,000) or glyceraldehyde-3-phosphate dehydrogenase (GAPDH, #CSA-335, Assay Designs, Ann Arbor, USA) (1:5000), were used as protein gel loading controls. Antibodies were diluted in Tris-buffered saline containing 0.5% Tween-20 and were incubated overnight at 4 °C, followed by horseradish peroxidase-conjugated secondary antibodies (Amersham Biosciences, Piscataway, NJ, USA) (1:2000) 1 h at RT. The blots were developed with a chemiluminescent substrate (Luminol 250 mM, Sigma). Band intensity was quantified by densitometry (Quantity One, Bio-Rad, Hercules, CA, USA).

### Nuclear extracts

Nuclear extracts were prepared from macrophage cultures. The cells we collected in Hepes buffer with protease and phosphatase inhibitors and were centrifuged at 300×*g* for 5 min at 4 °C. The pellet was suspended in hypotonic buffer (20 mM Hepes pH 7.5, 5 mM NaF, and 10 mM Na_2_MoO_4_). After 15 min at 4 °C, non-ionic detergent was added (50 μL Igepal 10%/mL), mixed and centrifuged at 12,000×*g* for 30 min. The pellet was suspended in 30 μL RIPA buffer. Twelve micrograms of nuclear protein was run in 10% polyacrylamide gels, and EP2 expression was analyzed by Western blotting as above. As gel loading control for nuclear protein, we used a mouse monoclonal antibody against TATA-binding protein (TBP, #ab51841, Abcam) diluted 1:500.

### Immunocytochemistry

The cells were seeded on polylysine-coated coverslips. The cells were fixed in 4% paraformaldehyde for 20 min, permeabilized with 0.2% Triton X-100 (Sigma) in PBS for 10 min, blocked with 3% goat serum in PBS for 1 h and incubated overnight at 4 °C with the primary rabbit antibodies against the EP2 (#APR-064, kindly provided by Alomone Labs) (1:100) and EP4 (#ab93486, Abcam, Cambridge, UK) (1:200) receptors. The next day, cells were washed and incubated with green fluorescence Alexa Fluor® 488 dye-labeled goat anti-rabbit IgG antibody (#A11070, Invitrogen, Glasgow, UK) for 1 h at room temperature. ToPro®-3 Iodide (T3605, Invitrogen) (1:1000) stained was performed to visualize the cell nuclei. The coverslips were mounted onto microscope slides using Fluormount-G® (Southern Biotech, Birmingham, AL, USA). Images were obtained with a laser confocal microscope (TCS SPE, Leica Microsystems, Wetzlar, Germany).

### Real-time RT-PCR

Total RNA was extracted using Purelink RNA Kit (Invitrogen). RNA quantity and purity were assessed in a ND-1000 micro-spectrophotometer (NanoDrop Technologies, Wilmington, DE, USA). One hundred fifty micrograms of total RNA was reverse-transcribed using a mixture of random primers (High Capacity cDNA Reverse Transcription kit, Applied Biosystems, Foster City, CA, USA). Real-time quantitative RT-PCR analysis was carried out by SYBR green I dye detection (#11761500, Invitrogen) or Taqman probes (#4304437, Life Technology, Carlsbad, CA, USA) using the iCycler iQTM Multicolor Real-Time Detection System (Bio-Rad). PCR primers were designed with Primer-Blast software of PubMed to bridge the exon-intron boundaries within the gene of interest to exclude amplification of contaminating genomic DNA. Primers (Table 1) were purchased from IDT (Conda, Spain) and Invitrogen. Optimized thermal cycling conditions for SYBR green assays were as follows 2 min at 50 °C, 10 min at 95 °C followed by 40 cycles of 15 sec at 95 °C and 1 min at 60 °C and finally 1 min at 95 °C, 1 min and 10 s at 55 °C. For Taqman system, the primers are listed in Table 1 and the qPCR conditions were 10 min at 95 °C followed by 40 cycles of 15 s at 95 °C and 1 min at 60 °C and finally 30 s at 40 °C. Data were collected after each cycle and were graphically displayed (iCycler iQTM Real-time Detection System Software, version 3.1, Bio-Rad). Melt curves were performed upon completion of the cycles to ensure absence of non-specific products. Quantification was performed by normalizing cycle threshold (Ct) values with the RPL14, GAPDH, or HPRT1 (Table 1) control gene Ct, and analysis was carried out with the 2−ΔΔCT method, as reported [7].

**Table 1:** List of primer sequences for mouse PCR

	Primer sequence 5′→3′	Accession no.	Amplicon length (bp)	Region
SYBR green				
TNF-α (Tnfa)	F: GGGGCCACCACGCTCTTCTGTC	NM_013693	155	Exon 1
	R: TGGGCTACGGGCTTGTCACTCG			Exon 3
Glucose-6P-dehydrogenase (G6pd)	F: CACAGTGGACGACATCCGAAA	NM_008062.2	103	Exon 4
	R: AGCTACATAGGAATTACGGGCAA			Exon 5
iNOS (Nos2)	F: CAGCTGGGCTGTACAAACCTT	NM_010927	95	Exon 17
	R: CATTGGAAGTGAAGCGTTTCG			Exon 18
VEGFA (Vegfa)	F: ATCTTCAAGCCGTCCTGTGTGC	NM_001025257.3	223	Exon 3
	R: TTGGCTTGTCACATTTTTCTGG			Exon 5/6
rpl14	F: GGCTTTAGTGGATGGACCCT	NM_025974	143	Exon 3
	R: ATTGATATCCGCCTTCTCCC			Exon 4
	ID Assay	Accession no.	Amplicon length (bp)	Exon boundary
Taqman probes				
EP1 (Ptger1)	Mm00443098_g1	NM_013641.2	85	Exon 2–3
EP2 (Ptger2)	Mm00436051_m1	NM_008964.4	73	Exon 1–2
EP3 (Ptger3)	Mm01316856_m1	NM_011196.2	79	Exon 1–2
EP4 (Ptger4)	Mm00436053_m1	NM_001136079.2	70	Exon 2–3
		NM_008965.2		
NOX2 (Cybb)	Mm01287743_m1	NM_007807.5	63	Exon 12–13
gapdh	Mm99999915_g1	NM_001289726.1	107	Exon 2–3
		NM_008084.3		
hprt1	Mm00446968_m1	NM_013556.2	65	Exon 6–7

### ELISA immunoassays

ELISA was used to measure the culture medium concentration of TNF-α (#88-7324-88, eBioscience, San Diego, CA, USA) and PGE_2_ (#900-001, Assay Designs, Ann Arbor, MI, USA).

### Nitrite assay

An indirect assessment of nitric oxide (NO) production was obtained by measuring NO decomposition products using the spectrophotometric assay based on the Griess reagent (1% sulfanilamide, 0.1% N-(1-naphthyl)-ethylenediamine dihydrochloride, and 5% phosphoric acid) [23]. Briefly, 50 μL of culture medium was collected 48 h after LPS treatment (100 ng/mL) in the presence of either the EP2 antagonist (PF 04418948, 1 μM) or the corresponding vehicle. Samples were incubated with equal volumes of Griess reagent for 10 min at room temperature. Optical density at 540 nm was determined using a microplate reader (Multiskan spectrum, Thermo Scientific, Waltham, MA, USA). Nitrite concentration was determined using a sodium nitrite standard curve.

### Statistical analyses

Comparison between two groups was carried out with the *t* test or the non-parametric Mann-Whitney test. One-way ANOVA or the Kruskal-Wallis test were used for multiple group comparisons, as appropriate, followed by the post hoc Bonferroni or Dunn test, respectively. Repeated measures design was applied when appropriate. Two-way ANOVA was used for grouped analyses. Statistical analyses were carried out using GraphPad software.

## Results

### COX-2 mediates the production of PGE_2_ in glial cells exposed to LPS

**Fig. 1:**
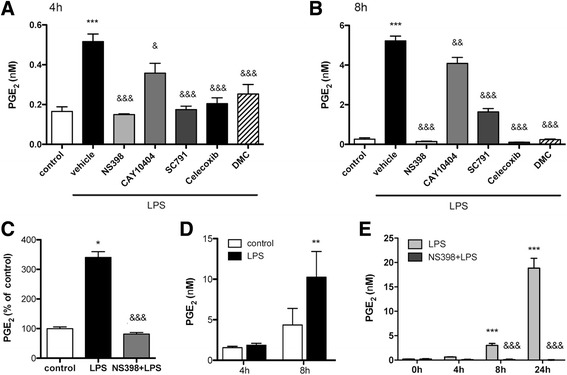
Cox-2 mediates LPS-induced PGE_2_ in astroglial and microglial cells. Concentration of PGE_2_ in the culture medium of astrocytes (**a**–**c**) and microglia (**d**, **e**), as determined by ELISA. **a**, **b** Reduction of LPS-induced PGE_2_ in the presence of various COX-2 inhibitors: NS-398 (3 μM), CAY10404 (10 nM), SC-791 (10 nM), celecoxib (10 μM), and the celecoxib analog DMC (10 μM), which does not inhibit COX-2 but interferes with mPGES-1 activity, 4 (**a**) and 8 h (**b**) after LPS exposure (*n* = 3–9 in two independent experiments). **c** The potent effect of NS-398 was validated in additional astroglia cultures 4 h after LPS (*n* = 3–4 replicates in three independent cultures). **d** Time-dependent production of PGE_2_ in microglia (*n* = 3–12 in three independent cultures). **e** NS-398 (3 μM) completely abrogates LPS-induced PGE_2_ production in microglia (*n* = 3–6 in two independent experiments). *p <0.05, **p < 0.01, ***p < 0.001 vs. control; &*p* < 0.05, &&*p* < 0.01, &&&*p* < 0.001 vs. LPS

LPS induces the production and release of PGE_2_ in glial cells [7]. The PGE_2_ concentration in the culture medium of astrocytes increased from basal levels of 0.9 ± 0.3 nM to 4.8 ± 3.1 nM (mean ± SD, in 11 independent experiments) 8 hours after LPS exposure, as assessed by ELISA assays. However, COX-2-deficient astrocytes do not produce PGE_2_ after LPS, as we previously reported [7]. Also, PGE_2_ production was completely abrogated by a range of COX-2 inhibitors (Fig. 1a, b). Notably, the compound dimethyl-celecoxib (DMC), an analog of celecoxib that does not have inhibitory effects on COX-2, also prevented the increase in PGE_2_ concentration after LPS (Fig. 1a, b), possibly due to its described inhibitory effects on microsomal PGE synthase-1 (mPGES-1) [24]. The potent effect of NS-398 was further validated in additional astroglia cultures (Fig. 1c) and was chosen to study COX-2 inhibition in purified microglia cultures. LPS-induced PGE_2_ in microglia (Fig. 1d) was also completely abrogated by the COX-2 inhibitor NS-398 (Fig. 1e). Altogether, the results showed that PGE_2_ production in response to LPS was mediated by the activity of induced COX-2 in glial cells.

### PGE_2_ attenuates the production of TNF-α induced by LPS

**Fig. 2:**
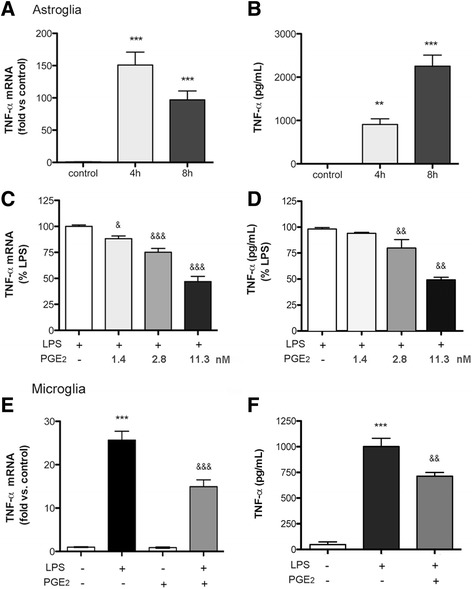
PGE_2_ attenuates the induction of TNF-α by LPS. The expression of TNF-α in astrocytes (**a**–**d**) and microglia (**e**, **f**) was studied by RT-PCR and ELISA 4 and 8 h after LPS exposure. **a**, **b** LPS induced the expression of TNF-α mRNA (*n* = 4 replicates per time point in three independent experiments) (**a**) and protein in the culture medium (*n* = 3–5 replicates in three independent experiments) (**b**). **c**, **d** Treatment with PGE_2_ 30 min before LPS reduces, in a dose-dependent manner (1.4–11.3 nM), the induction of TNF-α mRNA (**c**), and protein (**d**) (*n* = 3 replicates in three independent experiments). **e**, **f** In pure microglia cultures, treatment with PGE_2_ (11.3 nM) also reduced TNF-α mRNA expression (*n* = 3–6 replicates in three independent experiments) (**e**) and protein (*n* = 3–6 replicates in four independent experiments) (**f**) at 4 h. **p < 0.01, ***p < 0.001 vs. control; &*p* < 0.05, &&*p* < 0.01, &&&*p* < 0.001 vs. LPS

We then interrogated whether PGE_2_ could modulate the LPS-induced expression of tumor necrosis factor-α (TNF-α) (Fig. 2a, b). Treatment with exogenous PGE_2_ (1.4–11.3 nM) 30 min before LPS dose-dependently reduced the production of TNF-α messenger RNA (mRNA) and the release of TNF-α to the culture medium of astroglial (Fig. 2c, d) and microglial (Fig. 2e, f) cells.

### Naïve microglial cells express EP4 and EP2 receptors

**Fig. 3:**
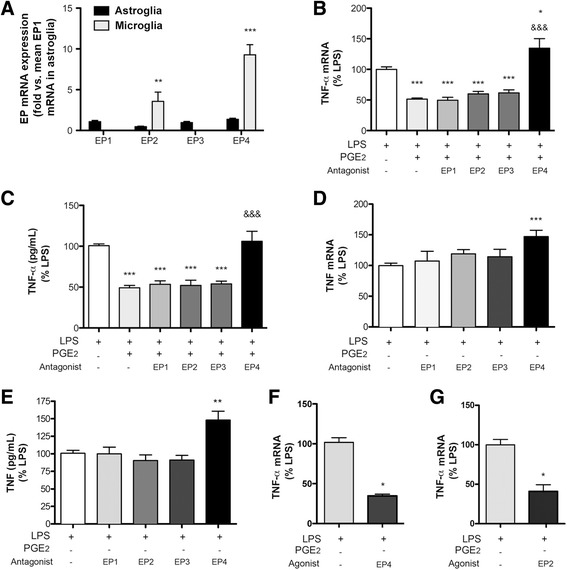
Relative EP mRNA expression in glial cells. **a** EP mRNA expression in naïve astroglia and microglia cultures (*n* = 6–7 in three independent cultures for each cell type). For comparative purposes, the level of mRNA for each EP and cell type is expressed as fold versus mean EP1 mRNA levels in astroglia. EP4 and to a lower extent EP2 expression is comparatively higher than EP1 and EP3 in naïve microglia and is higher in microglia than astroglia (**p < 0.01, ***p < 0.001). **b**–**g** The effect of EP drug antagonist and agonist on TNF-α mRNA expression (RT-PCR) and TNF-α concentration in the culture medium (ELISA) is shown for microglia cultures 8 h after LPS in the presence or absence of exogenous PGE_2_ (11.3 nM) (*n* = 6–9 in 2–3 independent cultures). EP1 antagonist (5 μM SC 51089), EP2 antagonist (1 μM PF 04418948), EP3 antagonist (0.5 μM L-798,106), and EP4 antagonist (1 μM GW 627368) were used. **b**, **c** The EP4 antagonist abrogates the PGE_2_-induced reduction of TNF-α mRNA (**b**) and protein (**c**) after LPS. **d**, **e** The EP4 antagonist increases TNF-α mRNA (**d**) and protein (**e**), as assessed by ELISA in the culture medium, in cells exposed to LPS in the absence of exogenous treatment with PGE_2_. **f**, **g** Treatment with an EP4 agonist (100 nM ONO-4819) (**f**) or an EP2 agonist (1 μM butaprost) (**g**) reduces LPS-induced TNF-α mRNA. (*n* = 3–4 independent cultures, three replicates in each). *p < 0.05, **p < 0.01, ***p < 0.001 vs. LPS, &&&*p* < 0.001 vs. LPS + PGE_2_

PGE_2_ induces selective responses depending on binding to four membrane receptors EP1-4, which trigger the activation of different intracellular signaling pathways. Thus, we examined the expression of EP receptor mRNA in our cultured astroglial and microglial cells. Naïve microglial cells predominantly expressed EP4 and to a lower extent EP2, whereas the level of expression of EP1 and EP3 was very low in these cells (Fig. 3a). In contrast, astrocytes showed similar levels of expression of all EP1-4 receptors with small differences (EP4 > EP1 > EP3 > EP2), and the levels of EP4 and EP2 mRNA were significantly lower than those in microglia (Fig. 3a).

We then used EP antagonists to identify the EP receptors functionally involved in the effect of PGE_2_ reducing the induction of TNF-α 8 h after LPS in microglia. The EP4 antagonist abrogated the PGE_2_-mediated reduction of LPS-induced TNF-α mRNA (Fig. 3b) and protein (Fig. 3c). However, EP1, EP2, or EP3 antagonists did not prevent the effect of PGE_2_ (Fig. 3b, c). We concluded that EP4 mediated the effect of PGE_2_ reducing LPS-induced TNF-α. In the absence of exogenous PGE_2_ treatment, the EP4 receptor antagonist also increased the expression of TNF-α mRNA (Fig. 3d) and protein (Fig. 3e) induced by LPS, suggesting that the natural production of PGE_2_ after LPS treatment down-regulated the expression of TNF-α via EP4. Accordingly, treatment with an EP4 agonist strongly reduced LPS-induced TNF-α (Fig. 3f). The EP2 agonist butaprost also reduced LPS-induced TNF-α mRNA (Fig. 3g), showing that activation of EP4 or EP2 receptors exert anti-inflammatory effects in naïve microglial cells. However, antagonizing EP2 did not significantly increase LPS-induced TNF-α (Fig. 3d, e) suggesting that the anti-inflammatory action of LPS-induced PGE_2_ occurred via EP4, in agreement with the higher affinity of EP4 than EP2 for PGE_2_ [25].

### LPS down-regulates EP4 and up-regulates EP2 expression during microglial activation

**Fig. 4:**
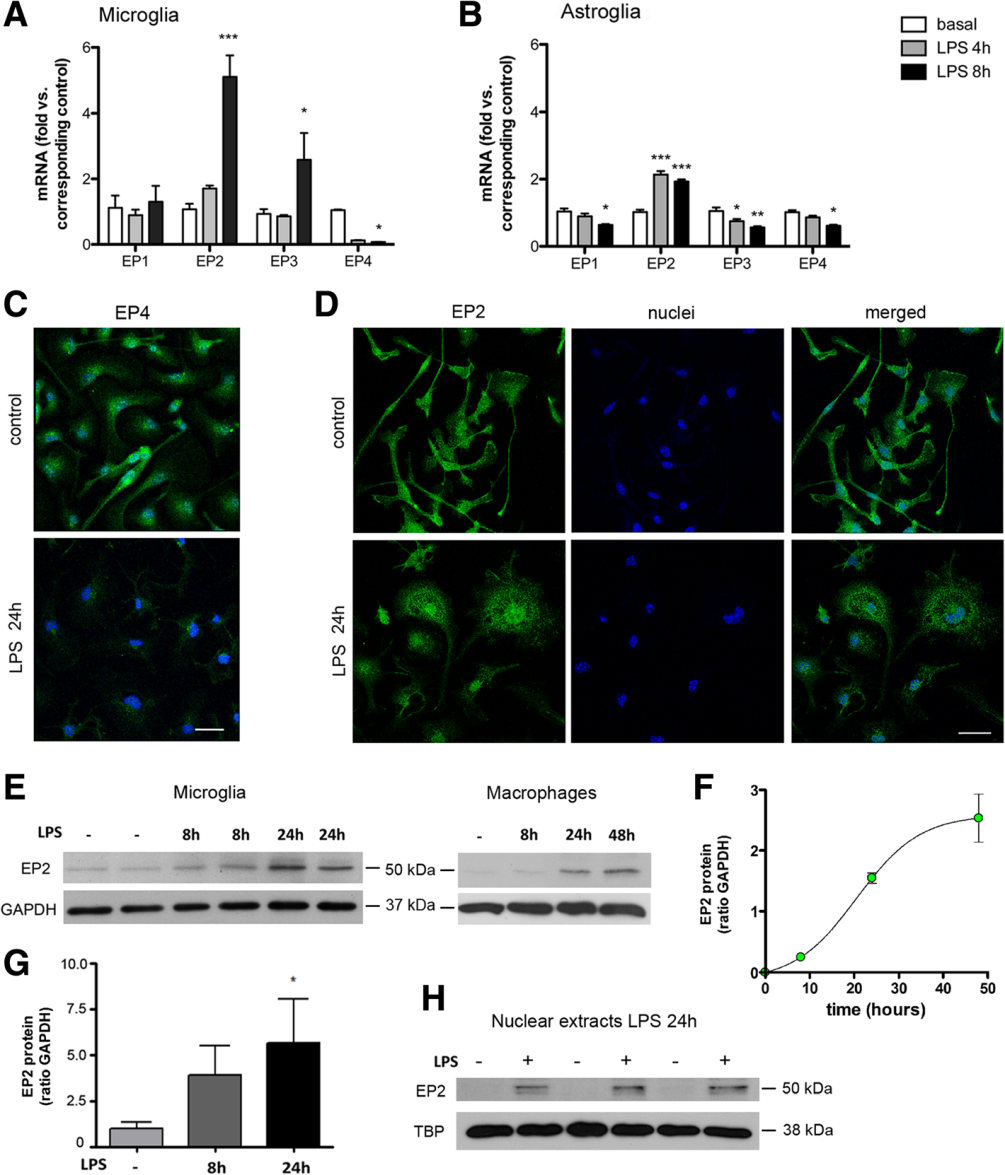
Effects of LPS on EP4 and EP2 mRNA expression. **a**, **b** LPS strongly depresses EP4 mRNA expression versus naïve cells, whereas it increases EP2 mRNA expression (*n* = 6–7 in three independent cultures for each cell type) in microglia (**a**) and astroglia (**b**) at 8 h. The level of mRNA for each EP and cell type is expressed as fold versus the corresponding basal mRNA levels in non-stimulated cells (control). **c** The intensity of microglia EP4 immunoreactivity is strongly reduced 24 h after LPS exposure (*n* = 4 in two independent experiments). **d** Microglia EP2 immunoreactivity is detected in a peri-nuclear/nuclear localization 24 h after LPS (*n* = 3 replicates per treatment group in three independent experiments). **e** Western blotting shows increased EP2 expression after LPS in microglia and macrophages. **f** Quantification of EP2 protein expression in control microglia and 8 and 24 h after LPS, where values (EP2/GAPDH ratio) are expressed as fold versus control (*p < 0.05, *n* = 3 replicates per time point in two independent experiments). **g** Quantification of EP2 protein (EP2/GAPDH ratio) in macrophages (*n* = 3 replicates in three experiments) also shows a progressive increase with time after LPS. Data were fit to a sigmoidal equation with non-linear regression analysis (*r* ^2^ = 0.83). **h** EP2 is found in the nuclear fraction of macrophages 24 h after LPS (three different cultures, *n* = 3 in each culture). *Scale bar*, 25 μm. **p <0.01, ***p <0.001 vs. control

We then examined whether LPS altered the expression of the EP receptors during the course of microglial activation. Notably, LPS significantly increased the expression of EP2 mRNA at 8 h, whereas it strongly down-regulated the expression of EP4 mRNA (Fig. 4a, b). The effects of LPS changing the level of expression of EP2 and EP4 receptor mRNA were larger in microglia than astroglia (Fig. 4a, b). In agreement to the mRNA changes, 24-h LPS reduced the microglial EP4 immunoreactivity (Fig. 4c), whereas strong EP2 immunoreactivity was detected (Fig. 4d). Western blotting revealed a significant increase in EP2 protein expression after LPS in microglia and macrophages (Fig. 4e–g). Furthermore, immunofluorescence showed nuclear/peri-nuclear EP2 immunoreactivity after classical microglial activation (Fig. 4d). To validate this finding, we obtained nuclear extracts of macrophages under control conditions and 24 h after LPS exposure. EP2 expression in the nuclear fraction was detected after LPS (Fig. 4h). Therefore, the cellular machinery to respond to PGE_2_ changed from naïve to primed cells, since the first predominantly expressed EP4, and to a lower extent EP2, whereas the latter mainly expressed EP2 that was prominent in nuclear/peri-nuclear zones.

### EP2 regulates the expression of genes and proteins involved in metabolism

**Fig. 5:**
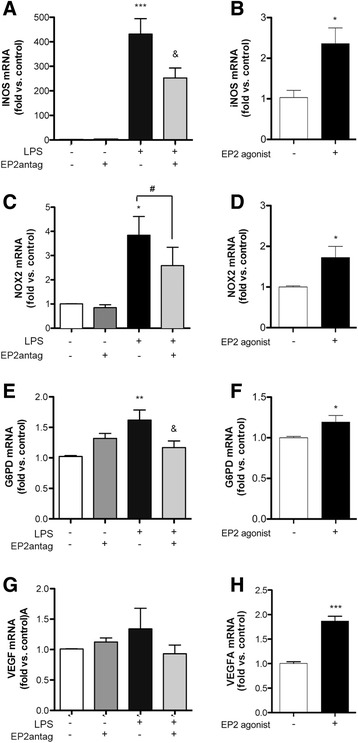
Involvement of EP2 in the expression of genes related to classical microglial activation. mRNA expression of iNOS (**a**, **b**), NOX2 (**c**, **d**), G6PD (**e**, **f**), and VEGFA (**g**, **h**) in microglia. **a**, **c**, **e**, **g** LPS increases mRNA expression at 24 h, and an EP2 antagonist (1 μM PF 04418948) reduces the effect of LPS (three to four independent experiments). **b**, **d**, **f**, **h** An EP2 agonist (1 μM butaprost) increases the mRNA expression of these genes at 8 h (three independent experiments). *p < 0.05, **p < 0.01, ***p < 0.001 vs. control; &*p* < 0.05 vs. LPS; ^#^ *p* = 0.06 vs. LPS. “EP2 antag” indicates EP2 antagonist

We then investigated whether EP2 was involved in classical microglia polarization by studying gene expression after 24 h in the presence or absence of EP2 antagonist drug. LPS induced the expression of the inducible nitric oxide synthase (iNOS) mRNA (Fig. 5a), a typical marker of classical macrophage activation. Previous studies reported that EP2 was involved in microglia iNOS expression [26, 27]. Accordingly, antagonizing EP2 reduced the expression of LPS-induced iNOS mRNA (Fig. 5a), whereas the EP2 agonist butaprost increased iNOS mRNA expression in naïve microglia (Fig. 5b). EP2 also showed a trend to reduce LPS-induced NADPH oxidase NOX2 mRNA (Fig. 5c), whereas the agonist increased NOX2 expression (Fig. 5d). Both NOX2 and iNOS enzymatic activities require NADPH oxidation. NADPH is produced through the pentose pathway that is involved in classical macrophage activation [28]. LPS increased the mRNA expression of glucose-6-phosphate dehydrogenase (G6PD) (Fig. 5e), the pentose pathway rate-limiting enzyme for NADPH production. The EP2 antagonist prevented this effect (Fig. 5e) whereas the EP2 agonist promoted G6PD mRNA expression (Fig. 5f). In addition, EP2 modulated the expression of the pro-angiogenic factor vascular endothelial growth factor (VEGFA) mRNA (Fig. 5g, h).

**Fig. 6:**
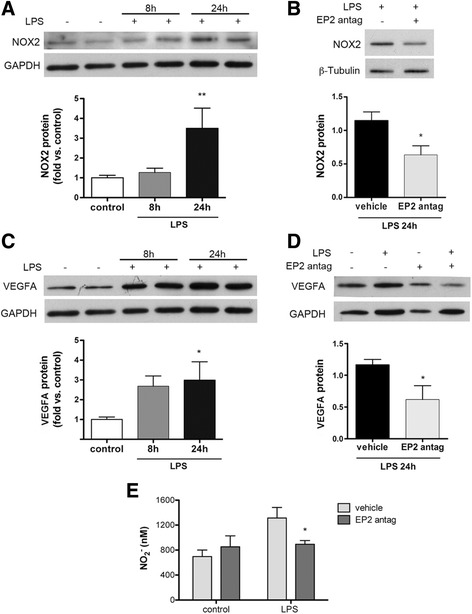
Effects of EP2 antagonist on LPS-induced protein expression in microglia. **a** LPS increases the expression of NOX2 protein at 24 h. **b** This effect is reduced by the EP2 antagonist (1 μM PF 04418948). **c** Also, LPS increases VEGFA protein expression and the EP2 antagonist attenuates this effect (**d**). **e** The EP2 antagonist reduces the nitrite production in the microglial culture medium induced by LPS at 48 h, indicating that EP2 contributes to LPS-induced iNOS activity. The figure illustrates a representative experiment (*n* = 7–11) out of the three independent experiments. **a**, **c** *n* = 3–6 in two to four independent experiments; **b**, **d** *n* = 3 obtained in three independent experiments. *p ≤ 0.05; **p < 0.01. “EP2 antag’ indicates EP2 antagonist

At the protein level, LPS increased the expression of NOX-2 at 24 h in microglia (Fig. 6a) (and also macrophages, shown in Additional file 3: Figure S3). The EP2 antagonist reduced LPS-induced NOX-2 in microglia (Fig. 6b), and similar effects were observed for VEGFA protein (Fig. 6c, d). Furthermore, the concentration of nitrites in the culture medium was measured as a surrogate marker of iNOS activity and nitric oxide production. The EP2 antagonist prevented the increase of the nitrite concentration induced by LPS at 48 h (Fig. 6e).

## Discussion

Inflammation induces the expression of COX-2 leading to the production of prostanoids that exert complex pleiotropic and cell type-dependent effects. COX-2 activity produces PGE_2_ in glial cells stimulated with LPS, and PGE_2_ exerts diverse actions depending on the activation of EP1–4 receptors. LPS-induced COX-2 expression and PGE_2_ production in astroglia and microglia but naïve microglial cells expressed higher levels of EP4 and EP2 than astrocytes. We then investigated the effects of PGE_2_ acting on EP2 and EP4 in microglia. In these cells, EP4 and EP2 receptors mediated certain anti-inflammatory actions of PGE_2_ since EP4 and EP2 agonists attenuated LPS-induced TNF-α production. However, the anti-inflammatory effect of PGE_2_ was mainly mediated through EP4 since only EP4 antagonists increased the production of LPS-induced TNF-α. The stronger effect of PGE_2_ on EP4 than EP2 was attributable to the comparatively greater affinity of EP4 for PGE_2_ [25] and to the higher expression of EP4 receptor in naïve microglial cells. However, LPS caused a potent reduction of EP4 expression while it increased the expression of EP2 at 24 and 48 h, showing a different regulation of the expression of the EP2 and EP4 genes. Furthermore, EP2 was detected in the nuclear fraction after classical microglial activation. Consequently, the response of primed microglial cells to PGE_2_ changed compared to that of naïve microglia due to differential EP receptor expression.

Previous reports showed that PGE_2_ reduces the expression of certain pro-inflammatory genes in microglia [19, 29]. EP4 receptor signaling attenuates brain inflammation [18] and exerts inhibitory effects on LPS-induced NFκB signaling [30]. However, EP4 activation promotes LPS-induced IL-23 secretion in immature dendritic cells and IL-17 production in activated T cells [31], and exerts pro-tumorigenic actions [32]. Therefore, modulation of EP4 activation in vivo can potentially exert benefits by attenuating the degree of innate immune responses in naïve microglia, but its various actions in different cell types could be detrimental or beneficial depending on the type and stage of disease. Likewise, EP2 activation is involved in a variety of physiopathological effects. For instance, PGE_2_ reduces amyloid β-induced phagocytosis in cultured rat microglia through its action on EP2 [43, 44]. EP2 regulates inflammatory responses in vivo since conditional deletion of EP2 in macrophages and microglia suppresses inflammation after systemic LPS administration to mice [45]. Also, EP2 promotes neutrophil recruitment through the local production of granulocyte colony-stimulating factor during acute inflammation [46] and mediates disease progression and inflammation in a model of amyotrophic lateral sclerosis [47]. In contrast, several studies attributed beneficial functions to EP2 since allosteric modulators of EP2 were protective in experimental models of excitotoxicity [48], EP2^−/−^ mice showed larger infarct volumes and worse neurological deficits compared to the wild-type mice after experimental brain ischemia [49, 50], and EP2-activation limited the synthesis of inflammatory mediators in a model of LPS-induced spinal cord inflammation [13]. Accordingly, in our study, agonizing EP2 in naïve microglia reduced the expression of TNF-α induced by exposure to LPS, in a similar way as EP4 did, but, in the absence of specific EP2 agonists, PGE_2_ mainly signaled through EP4. However, in primed microglia the expression of EP4 was strongly reduced whereas the expression of EP2 and, to a lower extent EP3, was up-regulated. While in this study, we focused on the effects of EP2 and EP4, further investigation is needed to find out the role of EP3 in primed microglial cells.

The presence of EP2 in peri-nuclear/nuclear locations suggests that activated microglia had an increased capacity to sense intracellular PGE_2_ that might signal to the cell nucleus. In previous studies EP receptors have been found in the nuclear membrane from where they modulate gene transcription [33, 34]. The present results show that EP2 is involved in the induction of VEGFA, the prototypical pro-angiogenic factor, and iNOS, which also modulates angiogenesis through the production of nitric oxide [35] by classically activated microglia. These findings agree with the reported reduction of iNOS expression by EP2 antagonization in the BV-2 microglia cell line exposed to hypoxia [27] and after genetic deletion of EP2 in microglia exposed to LPS [26]. Induction of iNOS is a typical hallmark of classical macrophage activation [36], which involves specific changes in the metabolic status of the cells [28]. iNOS catalyzes the production of nitric oxide from L-arginine using NADPH and oxygen [37]. NADPH is produced by the pentose monophosphate shunt after oxidation of glucose-6-phosphate by the rate-limiting enzyme G6PD, which is an important regulator of the cellular redox status [38, 39]. In turn, NADPH is oxidized by NADPH oxidases that generate oxygen reactive species [40]. Several lines of evidence suggest that G6PD might regulate iNOS expression [41] and angiogenesis [42]. Microglial activation with LPS increased the mRNA expression of G6PD and the NADPH oxidase NOX2, and again, our results point to the involvement of EP2 in these effects. These findings underscore a role of EP2 regulating metabolic processes participating in classical microglial activation.

**Fig. 7:**
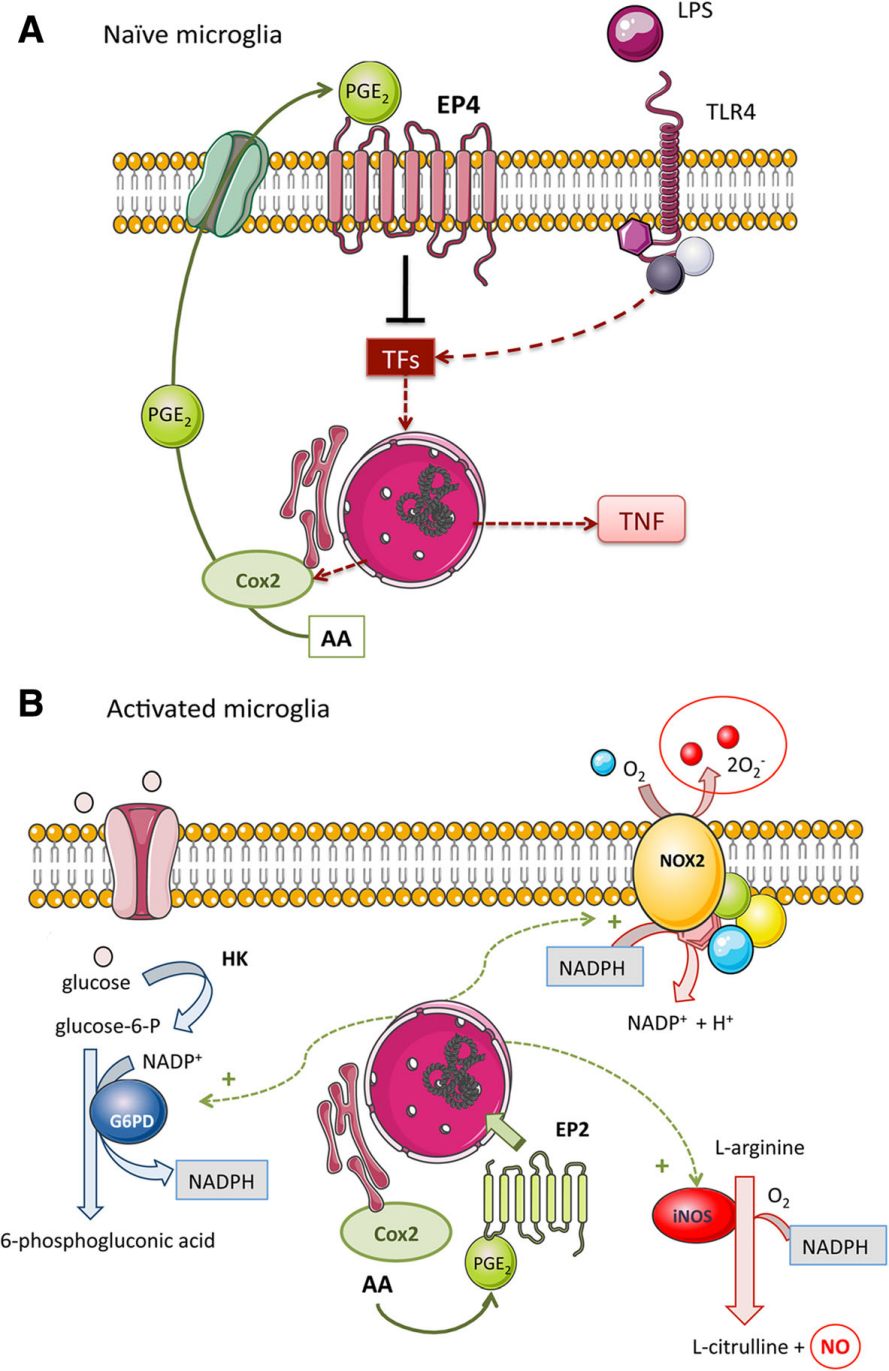
Schematic representation of the suggested dynamic effects of PGE_2_ on naïve and primed microglia. **a** Stimulation of naïve microglia with LPS activates TLR-4 leading to very rapid transcription of pro-inflammatory genes, including COX-2. COX-2 produces PGE_2_ and increases the concentration of PGE_2_ in the extracellular space. Although selective agonists of EP4 or EP2 exert anti-inflammatory effects, PGE_2_ mainly induces anti-inflammatory effects through EP4 in naïve microglia. **b** During the course of classical microglial activation, EP4 receptor expression is down-regulated whereas EP2 expression increases and is found in peri-nuclear/nuclear zones enabling responses to high intracellular PGE_2_. EP2 favors the induction of iNOS, NOX2, and G6PD. G6PD is involved in metabolic changes promoting glucose utilization through the pentose pathway that generates NADPH to fuel NO production by iNOS and free radical generation by NOX2. *AA* arachidonic acid, *COX2* cyclooxygenase-2, *G6PD* glucose-6-phosphate dehydrogenase, *HK* hexokinase, *TLR-4* Toll-like receptor-4, *TF* transcription factors, *TNF-α* tumor necrosis factor-α

Overall, the current findings suggest that EP4 and EP2 expression in naïve microglia mediates certain anti-inflammatory actions of PGE_2_, whereas EP2 expression in classically activated microglia regulates the expression of iNOS and facilitates a metabolic switch enabling glucose oxidation through the pentose monophosphate shunt. This pathway is known to generate the NADPH needed to fuel L-arginine metabolism through iNOS activity and sustain the activity of NADPH oxidases. According to these results, we suggest differential responses of naïve and classically activated microglia to PGE_2_ due to differential levels of EP2 and EP4 expression as well as different subcellular EP2 distribution, as schematically represented in Fig. 7.

## Conclusions

The present results highlight the key role of COX-2 in PGE_2_ production after an inflammatory challenge in glial cells where the COX-2/PGE_2_ axis exerts diverse effects mediated by dynamic changes in EP receptor expression. While PGE_2_ attenuates the expression of TNF-α by acting on EP4 in naïve microglia, EP4 expression decreases and EP2 expression increases in classically activated microglia where it senses high production of PGE_2_ and regulates crucial metabolic paths. These results suggest that adequate targeting of EP receptors in neuroinflammatory conditions would benefit from knowledge of the level of EP receptor expression during the course of the diseases.

## Additional files

Additional file 1: Figure S1. Illustration of microglia cultures. (TIF 473 kb)
Additional file 2: Figure S2. Exploratory dose-response experiments in macrophages and microglia. (TIF 918 kb)
Additional file 3: Figure S3. Time course of NOX2 protein expression after LPS in macrophages. (TIF 237 kb)

## Abbreviations

COX: Cyclooxygenase
DMSO: Dimethyl sulfoxide
EP: E-type prostanoid receptors
FBS: Fetal bovine serum
G6P: Glucose-6-phosphate
G6PD: Glucose-6-phosphate dehydrogenase
GAPDH: Glyceraldehyde-3-phosphate dehydrogenase
IL: Interleukin
iNOS: Inducible nitric oxide synthase
LPS: Bacterial lipopolysaccharide
mPGES-1: Microsomal PGE synthase-1
NOX2: NADPH oxidase 2
PBS: Phosphate-buffered saline
PGE_2_: Prostaglandin E_2_
TBP: TATA-binding protein
TLR-4: Toll-like receptor-4
TNF-α: Tumor necrosis factor-α
VEGFA: Vascular endothelial growth factor-A
