# Supplementary figures and images for: Differential expression of E-type prostanoid receptors 2 and 4 in microglia stimulated with lipopolysaccharide
Source: J Neuroinflammation. 2017 Jan 5;14:3. doi: 10.1186/s12974-016-0780-7 (PMC5234110; doi:10.1186/s12974-016-0780-7)

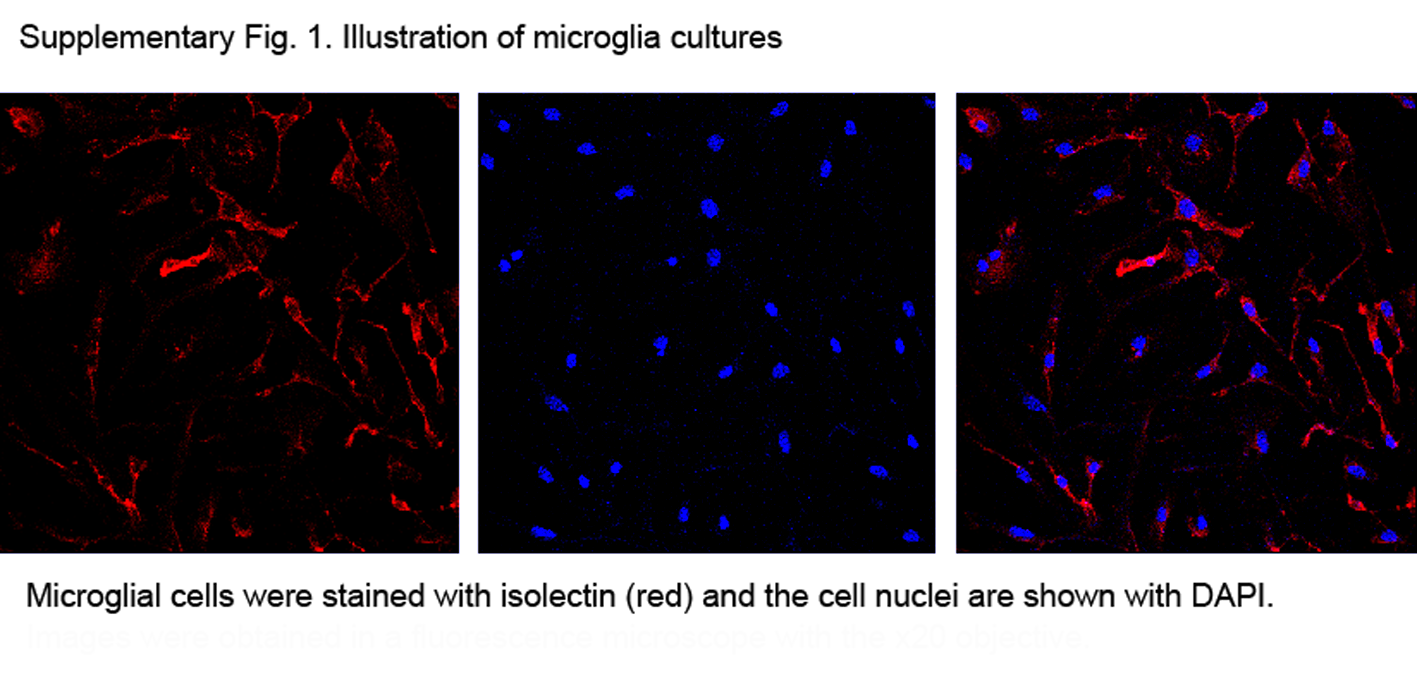

Supplement: Additional file 1: Figure S1. — Illustration of microglia cultures. (TIF 473 kb) [file 12974_2016_780_MOESM1_ESM.tif]

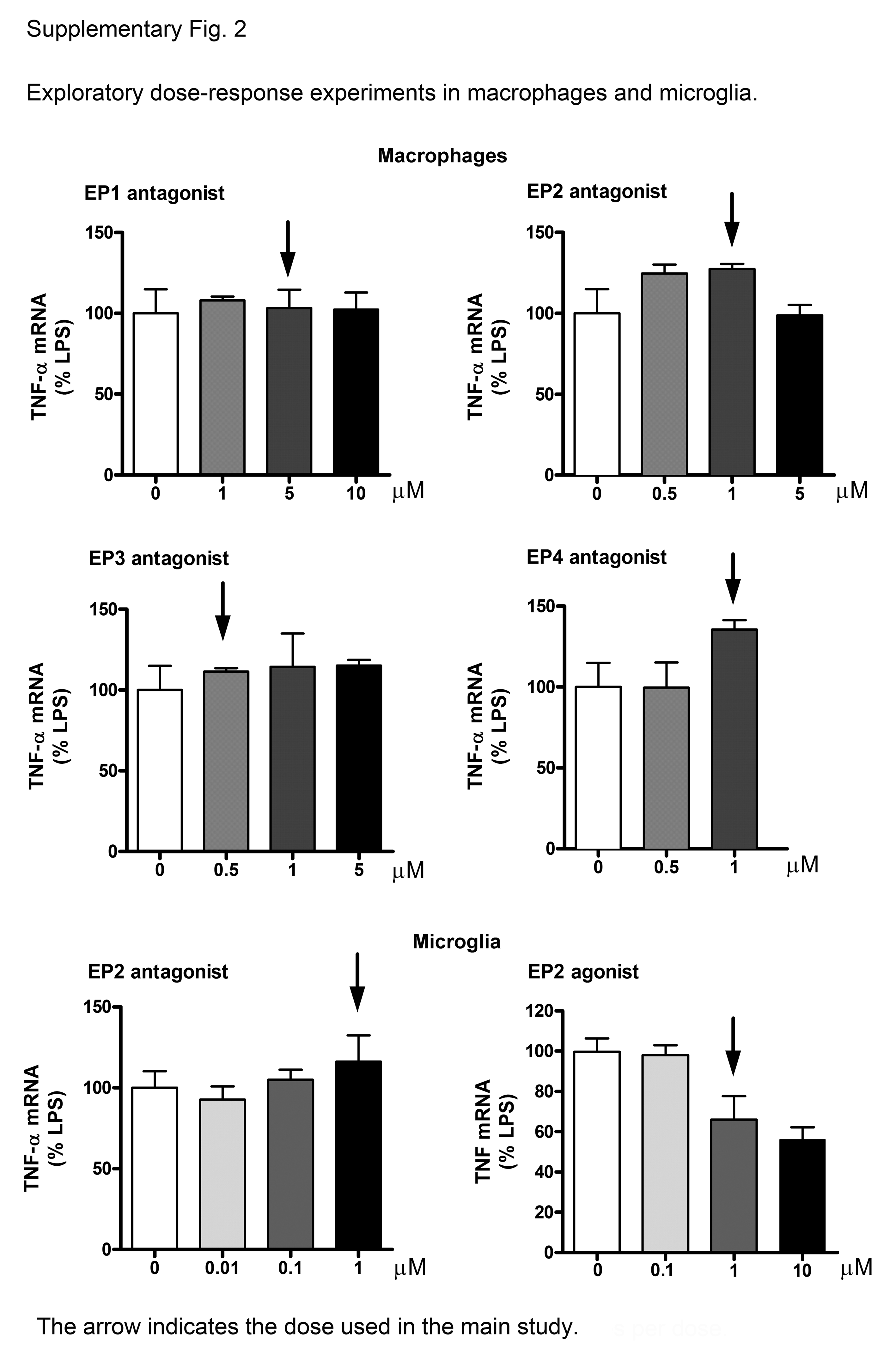

Supplement: Additional file 2: Figure S2. — Exploratory dose-response experiments in macrophages and microglia. (TIF 918 kb) [file 12974_2016_780_MOESM2_ESM.tif]

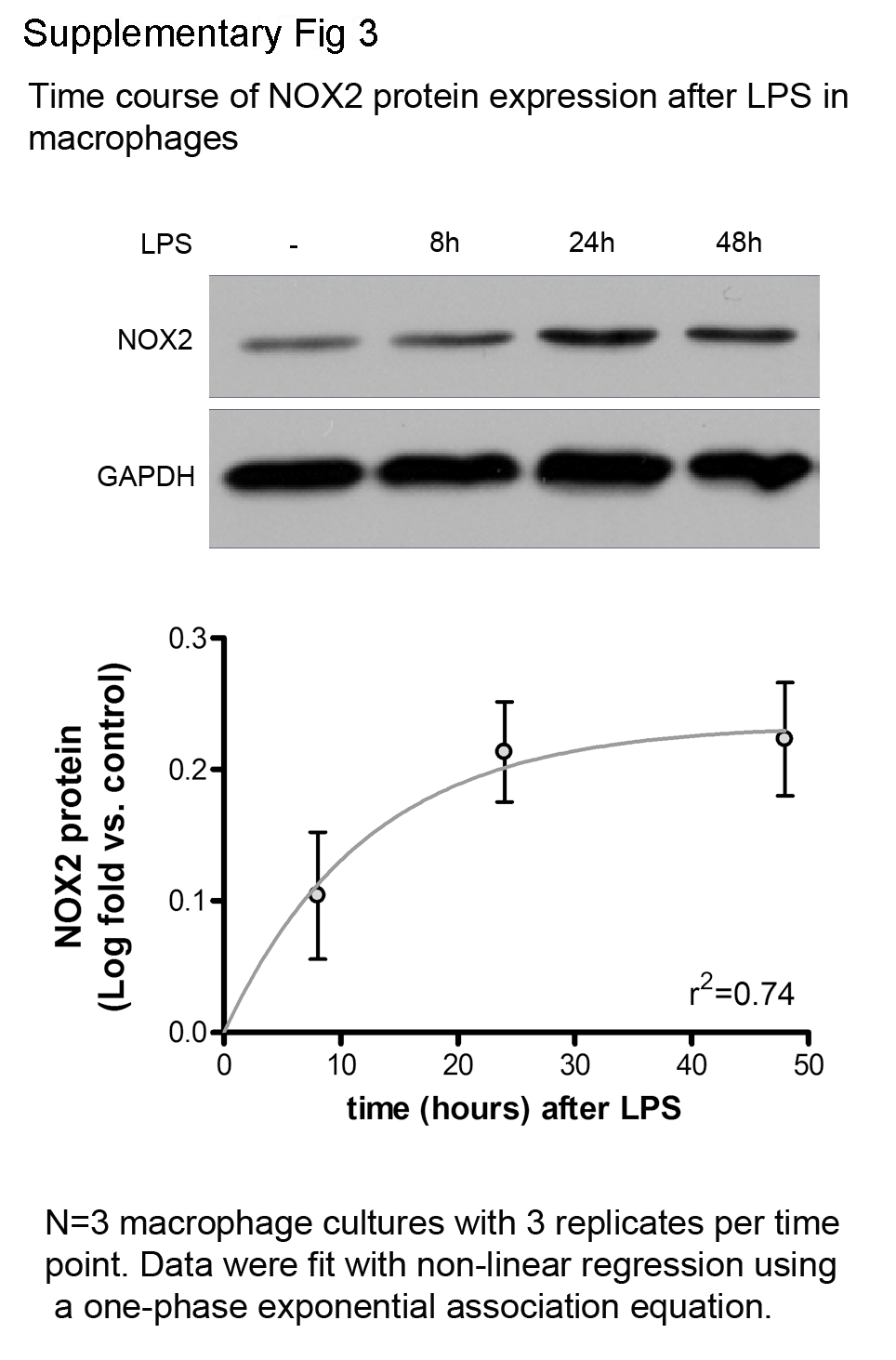

Supplement: Additional file 3: Figure S3. — Time course of NOX2 protein expression after LPS in macrophages. (TIF 237 kb) [file 12974_2016_780_MOESM3_ESM.tif]
